# 3-Hy­droxy-8-oxo-3-nor-methyl­chamigrane-2,7-peroxide

**DOI:** 10.1107/S1600536810024517

**Published:** 2010-06-30

**Authors:** Dongze Liu

**Affiliations:** a South China Sea Institute of Oceanology, Chinese Academy of Sciences, Guangzhou 510301, People’s Republic of China

## Abstract

In the title compound, C_14_H_22_O_4_ (systematic name: 9-hy­droxy-1,5,5-trimethyl-1,8-epidi­oxy­spiro­[5.5]decan-2-one),  which was isolated from the fermentation broth of *Steccherinum ochraceum*, the two six-membered rings adopt chair conformations and are bridged by a peroxide group. The hy­droxy H atom forms a three-centre cyclic inter­molecular O—H⋯(O,O′) hydrogen-bonding inter­action with a peroxide and a carbonyl O-atom acceptor, forming [100] chains.

## Related literature

For similar structures, see Miyashita *et al.* (1998 ▶).
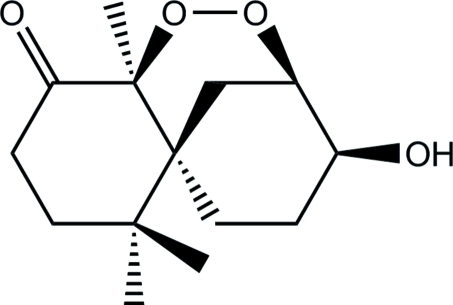


## Experimental

### 

#### Crystal data

C_14_H_22_O_4_
*M* *_r_* = 254.32
Orthorhombic, 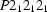

*a* = 7.3138 (4) Å
*b* = 12.4206 (7) Å
*c* = 13.9408 (8) Å
*V* = 1266.41 (12) Å^3^
*Z* = 4
Mo *K*α radiation
μ = 0.10 mm^−1^
*T* = 173 K
0.42 × 0.38 × 0.35 mm

#### Data collection

Bruker SMART 1000 CCD diffractometer
Absorption correction: multi-scan (*SADABS*; Sheldrick, 1996 ▶) *T*_min_ = 0.961, *T*_max_ = 0.967
6456 measured reflections
1602 independent reflections
1471 reflections with *I* > 2σ(*I*)
*R*_int_ = 0.021

#### Refinement

*R*[*F* ^2^ > 2σ(*F* ^2^)] = 0.037
*wR*(*F* ^2^) = 0.103
*S* = 1.08
1602 reflections
167 parameters
H-atom parameters constrained
Δρ_max_ = 0.30 e Å^−3^
Δρ_min_ = −0.17 e Å^−3^

### 

Data collection: *SMART* (Bruker, 2001 ▶); cell refinement: *SAINT-Plus* (Bruker, 2003 ▶); data reduction: *SAINT-Plus*; program(s) used to solve structure: *SHELXTL* (Sheldrick, 2008 ▶); program(s) used to refine structure: *SHELXTL*; molecular graphics: *SHELXTL*; software used to prepare material for publication: *SHELXTL*.

## Supplementary Material

Crystal structure: contains datablocks I, global. DOI: 10.1107/S1600536810024517/zs2044sup1.cif

Structure factors: contains datablocks I. DOI: 10.1107/S1600536810024517/zs2044Isup2.hkl

Additional supplementary materials:  crystallographic information; 3D view; checkCIF report

## Figures and Tables

**Table 1:** Hydrogen-bond geometry (Å, °)

*D*—H⋯*A*	*D*—H	H⋯*A*	*D*⋯*A*	*D*—H⋯*A*
O2—H2*A*⋯O1^i^	0.84	2.51	3.112 (2)	130
O2—H2*A*⋯O4^i^	0.84	2.17	2.943 (2)	154

Symmetry code: (i) 
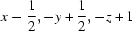
.

**Fig. 1.**
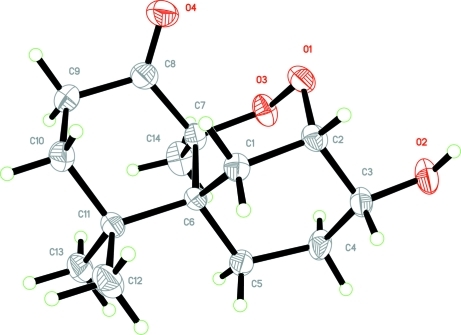
Molecular configuration and atom numbering scheme for (I), with displacement ellipsoids for non-H atoms drawn at the 50% probability level.

## supplementary crystallographic information

### Comment

Mushrooms have proved to be a rich source of secondary metabolites with unusual structures as well as interesting biological activities. As a continuation of our study characterizing bioactive metabolites from higher fungi in China, a new norsesquiterpene peroxide, 3-hydroxy-8-oxo-3-nor-methylchamigrane-2,7-peroxide, C_14_H_22_O_4_ (I) was isolated from the fermentation broth of *Steccherinum ochraceum*. The six-membered C1–C2–C3–C4–C5–C6 and C1–C2–O(1)–O(2)–C7–C6 rings both adopt chair conformations and are bridged by the peroxide group (Fig. 1). The absolute configuration was not determined for the four chiral centres in this compound and the structures of no other compounds with a similar peroxide-bridged cage system are present in the CSD. The hydroxyl H in (I) gives a three-centre cyclic intermolecular O–H···O,O' hydrogen-bonding interaction with both a peroxide and a carbonyl O acceptor, (Table 1) giving one-dimensional chains which extend down the *a* direction of the unit cell.

### Experimental

*S. ochraceum* was collected from the Ailao Mountain of Yunnan Province, China. The strain was cultured in a liquid medium composed of potato (200 g), glucose (20 g), KH_2_PO_4_ (3 g), MgSO_4_ (1.5 g), citric acid (0.1 g), and thiamin hydrochloride (10 mg) in 1 L of deionized water. The fungus was grown in reagent bottles (500 mL; media of 300 mL). The pH was adjusted to 6.5 before autoclaving. Fermentation was carried out on a shaker at 22° C and 150 RPM for 25 days. The culture broth (20 L) was filtered to remove the mycelium. The filtrate was then successively extracted twice with ethyl acetate, and the crude extract (3.5 g) was chromatographed on a silica gel column using a CHCl_3_/MeOH gradient. Several fractions of increasing polarity were collected. Fraction II (850 mg) eluted with CHCl_3_/MeOH (100:1, v/v) was subjected to column chromatography over silica gel and Sephadex LH-20, using a petroleum ether/ethyl acetate (8:1, v/v) and CHCl_3_/MeOH (1:1, v/v) eluents respectively, and further purified by repeated recrystallization from MeOH to yield (I) (150 mg). Single crystals were obtained by slow evaporation of a MeOH solution.

### Refinement

All H atoms were placed in calculated sites and allowed to ride with C–H = 0.98–1.00 Å and O–H = 0.84 Å and *U*_iso_ = 1.2*U*_eq_C(methylene) and O(hydroxyl) or 1.5*U*_eq_C(methyl). The absolute configuration could not be determined for this light atom compound and Friedel pairs were averaged in the final refinement with the configuration for the four chiral centres as 2*S*,3*R*,6*S*,7*S*.

### Crystal data

**Table:**

C_14_H_22_O_4_	*F*(000) = 552
*M**_r_* = 254.32	*D*_x_ = 1.334 Mg m^−^^3^
Orthorhombic, *P*2_1_2_1_2_1_	Mo *K*α radiation, λ = 0.71073 Å
Hall symbol: P 2ac 2ab	Cell parameters from 4034 reflections
*a* = 7.3138 (4) Å	θ = 2.8–27.0°
*b* = 12.4206 (7) Å	µ = 0.10 mm^−^^1^
*c* = 13.9408 (8) Å	*T* = 173 K
*V* = 1266.41 (12) Å^3^	Block, colorless
*Z* = 4	0.42 × 0.38 × 0.35 mm

### Data collection

**Table:**

Bruker SMART 1000 CCD diffractometer	1602 independent reflections
Radiation source: fine-focus sealed tube	1471 reflections with *I* > 2σ(*I*)
graphite	*R*_int_ = 0.021
ω scans	θ_max_ = 27.0°, θ_min_ = 2.2°
Absorption correction: multi-scan (*SADABS*; Sheldrick, 1996)	*h* = −5→9
*T*_min_ = 0.961, *T*_max_ = 0.967	*k* = −15→15
6456 measured reflections	*l* = −17→13

### Refinement

**Table:**

Refinement on *F*^2^	Primary atom site location: structure-invariant direct methods
Least-squares matrix: full	Secondary atom site location: difference Fourier map
*R*[*F*^2^ > 2σ(*F*^2^)] = 0.037	Hydrogen site location: inferred from neighbouring sites
*wR*(*F*^2^) = 0.103	H-atom parameters constrained
*S* = 1.08	*w* = 1/[σ^2^(*F*_o_^2^) + (0.0611*P*)^2^ + 0.2837*P*] where *P* = (*F*_o_^2^ + 2*F*_c_^2^)/3
1602 reflections	(Δ/σ)_max_ < 0.001
167 parameters	Δρ_max_ = 0.30 e Å^−^^3^
0 restraints	Δρ_min_ = −0.17 e Å^−^^3^

### Special details

**Table:**

Geometry. All e.s.d.'s (except the e.s.d. in the dihedral angle between two l.s. planes) are estimated using the full covariance matrix. The cell e.s.d.'s are taken into account individually in the estimation of e.s.d.'s in distances, angles and torsion angles; correlations between e.s.d.'s in cell parameters are only used when they are defined by crystal symmetry. An approximate (isotropic) treatment of cell e.s.d.'s is used for estimating e.s.d.'s involving l.s. planes.
Refinement. Refinement of *F*^2^ against ALL reflections. The weighted *R*-factor *wR* and goodness of fit *S* are based on *F*^2^, conventional *R*-factors *R* are based on *F*, with *F* set to zero for negative *F*^2^. The threshold expression of *F*^2^ > σ(*F*^2^) is used only for calculating *R*-factors(gt) *etc*. and is not relevant to the choice of reflections for refinement. *R*-factors based on *F*^2^ are statistically about twice as large as those based on *F*, and *R*- factors based on ALL data will be even larger.

### Fractional atomic coordinates and isotropic or equivalent isotropic displacement parameters (Å^2^)

**Table:**

	*x*	*y*	*z*	*U*_iso_*/*U*_eq_	
C1	0.4858 (3)	0.29411 (15)	0.68490 (13)	0.0218 (4)	
H1A	0.5496	0.2309	0.7124	0.026*	
H1B	0.3532	0.2857	0.6971	0.026*	
C2	0.5204 (3)	0.29930 (15)	0.57779 (13)	0.0225 (4)	
H2	0.4747	0.2304	0.5495	0.027*	
C3	0.4112 (3)	0.39043 (16)	0.53239 (13)	0.0247 (4)	
H3	0.2786	0.3719	0.5380	0.030*	
C4	0.4405 (3)	0.49914 (16)	0.58127 (15)	0.0296 (5)	
H4A	0.3429	0.5494	0.5608	0.036*	
H4B	0.5591	0.5295	0.5602	0.036*	
C5	0.4393 (3)	0.49050 (15)	0.69173 (14)	0.0259 (4)	
H5A	0.3114	0.4812	0.7134	0.031*	
H5B	0.4846	0.5593	0.7187	0.031*	
C6	0.5554 (2)	0.39771 (14)	0.73338 (13)	0.0183 (4)	
C7	0.7632 (3)	0.40990 (16)	0.70409 (13)	0.0229 (4)	
C8	0.8819 (3)	0.32554 (18)	0.75389 (15)	0.0280 (4)	
C9	0.8623 (3)	0.3215 (2)	0.86128 (15)	0.0334 (5)	
H9A	0.9072	0.3896	0.8897	0.040*	
H9B	0.9367	0.2617	0.8873	0.040*	
C10	0.6617 (3)	0.30478 (17)	0.88798 (14)	0.0285 (4)	
H10A	0.6507	0.3054	0.9588	0.034*	
H10B	0.6229	0.2328	0.8653	0.034*	
C11	0.5305 (3)	0.38957 (16)	0.84637 (14)	0.0221 (4)	
C12	0.3354 (3)	0.3526 (2)	0.87222 (16)	0.0331 (5)	
H12A	0.3233	0.3480	0.9421	0.050*	
H12B	0.3122	0.2817	0.8438	0.050*	
H12C	0.2467	0.4046	0.8472	0.050*	
C13	0.5579 (3)	0.49723 (17)	0.89893 (14)	0.0312 (5)	
H13A	0.6821	0.5238	0.8871	0.047*	
H13B	0.5401	0.4866	0.9679	0.047*	
H13C	0.4691	0.5499	0.8752	0.047*	
C14	0.8524 (3)	0.52104 (18)	0.72087 (16)	0.0341 (5)	
H14A	0.7730	0.5775	0.6949	0.051*	
H14B	0.9713	0.5235	0.6885	0.051*	
H14C	0.8695	0.5326	0.7898	0.051*	
O1	0.7133 (2)	0.30323 (12)	0.56148 (10)	0.0295 (4)	
O2	0.4515 (2)	0.40426 (12)	0.43304 (10)	0.0333 (4)	
H2A	0.4353	0.3457	0.4041	0.050*	
O3	0.7841 (2)	0.40379 (13)	0.60137 (10)	0.0298 (3)	
O4	0.9872 (2)	0.26855 (15)	0.71020 (12)	0.0423 (5)	

### Atomic displacement parameters (Å^2^)

**Table:**

	*U*^11^	*U*^22^	*U*^33^	*U*^12^	*U*^13^	*U*^23^
C1	0.0262 (9)	0.0173 (8)	0.0219 (9)	−0.0021 (8)	−0.0017 (7)	−0.0004 (7)
C2	0.0273 (9)	0.0180 (8)	0.0222 (9)	−0.0018 (8)	−0.0007 (8)	−0.0047 (7)
C3	0.0310 (10)	0.0245 (9)	0.0187 (8)	−0.0002 (8)	−0.0046 (8)	−0.0016 (7)
C4	0.0439 (12)	0.0208 (9)	0.0242 (9)	0.0050 (9)	−0.0104 (9)	−0.0004 (8)
C5	0.0338 (10)	0.0211 (9)	0.0229 (9)	0.0069 (9)	−0.0053 (8)	−0.0041 (8)
C6	0.0198 (8)	0.0179 (8)	0.0174 (8)	−0.0001 (7)	0.0005 (7)	−0.0018 (7)
C7	0.0234 (9)	0.0278 (9)	0.0174 (8)	−0.0029 (8)	−0.0002 (7)	−0.0005 (8)
C8	0.0204 (9)	0.0350 (10)	0.0284 (10)	0.0006 (9)	−0.0018 (8)	−0.0055 (9)
C9	0.0306 (11)	0.0433 (12)	0.0264 (10)	0.0114 (10)	−0.0051 (9)	0.0013 (10)
C10	0.0342 (11)	0.0295 (10)	0.0217 (9)	0.0024 (9)	0.0005 (8)	0.0035 (8)
C11	0.0228 (9)	0.0254 (9)	0.0182 (8)	0.0007 (8)	0.0011 (7)	−0.0024 (8)
C12	0.0274 (10)	0.0467 (13)	0.0251 (10)	−0.0053 (10)	0.0068 (8)	−0.0040 (9)
C13	0.0389 (12)	0.0318 (10)	0.0228 (9)	0.0023 (10)	−0.0015 (9)	−0.0080 (8)
C14	0.0363 (11)	0.0364 (11)	0.0297 (10)	−0.0155 (10)	0.0007 (9)	0.0004 (9)
O1	0.0286 (7)	0.0345 (8)	0.0253 (7)	0.0029 (6)	0.0013 (6)	−0.0095 (6)
O2	0.0511 (10)	0.0305 (7)	0.0184 (6)	−0.0015 (8)	−0.0050 (7)	−0.0015 (6)
O3	0.0307 (7)	0.0378 (8)	0.0208 (7)	−0.0093 (7)	0.0037 (6)	−0.0027 (6)
O4	0.0329 (8)	0.0555 (10)	0.0386 (9)	0.0160 (8)	−0.0052 (7)	−0.0158 (8)

### Geometric parameters (Å, °)

**Table:**

C1—C2	1.516 (3)	C8—O4	1.211 (3)
C1—C6	1.540 (2)	C8—C9	1.505 (3)
C1—H1A	0.9900	C9—C10	1.528 (3)
C1—H1B	0.9900	C9—H9A	0.9900
C2—O1	1.430 (2)	C9—H9B	0.9900
C2—C3	1.523 (3)	C10—C11	1.538 (3)
C2—H2	1.0000	C10—H10A	0.9900
C3—O2	1.426 (2)	C10—H10B	0.9900
C3—C4	1.528 (3)	C11—C13	1.538 (3)
C3—H3	1.0000	C11—C12	1.542 (3)
C4—C5	1.544 (3)	C12—H12A	0.9800
C4—H4A	0.9900	C12—H12B	0.9800
C4—H4B	0.9900	C12—H12C	0.9800
C5—C6	1.545 (3)	C13—H13A	0.9800
C5—H5A	0.9900	C13—H13B	0.9800
C5—H5B	0.9900	C13—H13C	0.9800
C6—C7	1.581 (3)	C14—H14A	0.9800
C6—C11	1.589 (2)	C14—H14B	0.9800
C7—O3	1.442 (2)	C14—H14C	0.9800
C7—C8	1.527 (3)	O1—O3	1.462 (2)
C7—C14	1.545 (3)	O2—H2A	0.8400
			
C2—C1—C6	109.98 (15)	O4—C8—C9	122.8 (2)
C2—C1—H1A	109.7	O4—C8—C7	122.27 (19)
C6—C1—H1A	109.7	C9—C8—C7	114.91 (18)
C2—C1—H1B	109.7	C8—C9—C10	109.78 (18)
C6—C1—H1B	109.7	C8—C9—H9A	109.7
H1A—C1—H1B	108.2	C10—C9—H9A	109.7
O1—C2—C1	108.82 (16)	C8—C9—H9B	109.7
O1—C2—C3	115.22 (17)	C10—C9—H9B	109.7
C1—C2—C3	110.70 (16)	H9A—C9—H9B	108.2
O1—C2—H2	107.3	C9—C10—C11	114.46 (17)
C1—C2—H2	107.3	C9—C10—H10A	108.6
C3—C2—H2	107.3	C11—C10—H10A	108.6
O2—C3—C2	112.61 (17)	C9—C10—H10B	108.6
O2—C3—C4	107.32 (16)	C11—C10—H10B	108.6
C2—C3—C4	113.44 (16)	H10A—C10—H10B	107.6
O2—C3—H3	107.7	C13—C11—C10	109.54 (16)
C2—C3—H3	107.7	C13—C11—C12	105.55 (17)
C4—C3—H3	107.7	C10—C11—C12	106.57 (17)
C3—C4—C5	112.51 (16)	C13—C11—C6	113.71 (16)
C3—C4—H4A	109.1	C10—C11—C6	110.25 (15)
C5—C4—H4A	109.1	C12—C11—C6	110.88 (16)
C3—C4—H4B	109.1	C11—C12—H12A	109.5
C5—C4—H4B	109.1	C11—C12—H12B	109.5
H4A—C4—H4B	107.8	H12A—C12—H12B	109.5
C4—C5—C6	115.08 (16)	C11—C12—H12C	109.5
C4—C5—H5A	108.5	H12A—C12—H12C	109.5
C6—C5—H5A	108.5	H12B—C12—H12C	109.5
C4—C5—H5B	108.5	C11—C13—H13A	109.5
C6—C5—H5B	108.5	C11—C13—H13B	109.5
H5A—C5—H5B	107.5	H13A—C13—H13B	109.5
C1—C6—C5	106.09 (15)	C11—C13—H13C	109.5
C1—C6—C7	106.51 (15)	H13A—C13—H13C	109.5
C5—C6—C7	111.08 (16)	H13B—C13—H13C	109.5
C1—C6—C11	110.13 (15)	C7—C14—H14A	109.5
C5—C6—C11	110.94 (15)	C7—C14—H14B	109.5
C7—C6—C11	111.84 (15)	H14A—C14—H14B	109.5
O3—C7—C8	110.81 (16)	C7—C14—H14C	109.5
O3—C7—C14	98.79 (15)	H14A—C14—H14C	109.5
C8—C7—C14	107.71 (16)	H14B—C14—H14C	109.5
O3—C7—C6	110.67 (15)	C2—O1—O3	108.54 (14)
C8—C7—C6	111.29 (16)	C3—O2—H2A	109.5
C14—C7—C6	116.91 (17)	C7—O3—O1	112.66 (14)
			
C6—C1—C2—O1	63.6 (2)	C6—C7—C8—O4	128.6 (2)
C6—C1—C2—C3	−63.9 (2)	O3—C7—C8—C9	−176.93 (18)
O1—C2—C3—O2	50.9 (2)	C14—C7—C8—C9	76.0 (2)
C1—C2—C3—O2	174.94 (16)	C6—C7—C8—C9	−53.3 (2)
O1—C2—C3—C4	−71.2 (2)	O4—C8—C9—C10	−126.6 (2)
C1—C2—C3—C4	52.8 (2)	C7—C8—C9—C10	55.4 (3)
O2—C3—C4—C5	−168.47 (18)	C8—C9—C10—C11	−56.0 (3)
C2—C3—C4—C5	−43.4 (3)	C9—C10—C11—C13	−72.0 (2)
C3—C4—C5—C6	46.4 (3)	C9—C10—C11—C12	174.30 (18)
C2—C1—C6—C5	63.35 (19)	C9—C10—C11—C6	53.9 (2)
C2—C1—C6—C7	−55.08 (19)	C1—C6—C11—C13	−167.83 (16)
C2—C1—C6—C11	−176.53 (15)	C5—C6—C11—C13	−50.7 (2)
C4—C5—C6—C1	−55.3 (2)	C7—C6—C11—C13	73.9 (2)
C4—C5—C6—C7	60.0 (2)	C1—C6—C11—C10	68.7 (2)
C4—C5—C6—C11	−174.90 (17)	C5—C6—C11—C10	−174.15 (16)
C1—C6—C7—O3	52.6 (2)	C7—C6—C11—C10	−49.5 (2)
C5—C6—C7—O3	−62.5 (2)	C1—C6—C11—C12	−49.1 (2)
C11—C6—C7—O3	172.94 (15)	C5—C6—C11—C12	68.1 (2)
C1—C6—C7—C8	−71.10 (18)	C7—C6—C11—C12	−167.31 (16)
C5—C6—C7—C8	173.81 (15)	C1—C2—O1—O3	−64.81 (18)
C11—C6—C7—C8	49.3 (2)	C3—C2—O1—O3	60.17 (19)
C1—C6—C7—C14	164.57 (16)	C8—C7—O3—O1	66.13 (19)
C5—C6—C7—C14	49.5 (2)	C14—C7—O3—O1	178.97 (15)
C11—C6—C7—C14	−75.1 (2)	C6—C7—O3—O1	−57.8 (2)
O3—C7—C8—O4	5.0 (3)	C2—O1—O3—C7	64.05 (18)
C14—C7—C8—O4	−102.0 (2)		

### Hydrogen-bond geometry (Å, °)

**Table:**

*D*—H···*A*	*D*—H	H···*A*	*D*···*A*	*D*—H···*A*
O2—H2A···O1^i^	0.84	2.51	3.112 (2)	130
O2—H2A···O4^i^	0.84	2.17	2.943 (2)	154
C2—H2···O1^i^	1.00	2.49	3.231 (2)	130

Symmetry codes: (i) *x*−1/2, −*y*+1/2, −*z*+1.
